# QTL Mapping for Pest and Disease Resistance in Cassava and Coincidence of Some QTL with Introgression Regions Derived from *Manihot glaziovii*

**DOI:** 10.3389/fpls.2017.01168

**Published:** 2017-07-21

**Authors:** Inosters Nzuki, Manpreet S. Katari, Jessen V. Bredeson, Esther Masumba, Fortunus Kapinga, Kasele Salum, Geoffrey S. Mkamilo, Trushar Shah, Jessica B. Lyons, Daniel S. Rokhsar, Steve Rounsley, Alexander A. Myburg, Morag E. Ferguson

**Affiliations:** ^1^Department of Genetics, Forestry and Agricultural Biotechnology Institute (FABI), University of Pretoria Pretoria, South Africa; ^2^International Institute of Tropical Agriculture Nairobi, Kenya; ^3^Department of Biology, New York University New York, NY, United States; ^4^Molecular and Cell Biology Department, University of California, Berkeley Berkeley, CA, United States; ^5^Sugarcane Research Institute Kibaha, Tanzania; ^6^Naliendele Agricultural Research Institute Mtwara, Tanzania; ^7^Lake Zone Agricultural Research and Development Institute Mwanza, Tanzania; ^8^Genus plc DeForest, WI, United States

**Keywords:** quantitative trait loci, introgression, cassava brown streak disease, genetic linkage map, Kiroba

## Abstract

Genetic mapping of quantitative trait loci (QTL) for resistance to cassava brown streak disease (CBSD), cassava mosaic disease (CMD), and cassava green mite (CGM) was performed using an F_1_ cross developed between the Tanzanian landrace, Kiroba, and a breeding line, AR37-80. The population was evaluated for two consecutive years in two sites in Tanzania. A genetic linkage map was derived from 106 F_1_ progeny and 1,974 SNP markers and spanned 18 chromosomes covering a distance of 1,698 cM. Fifteen significant QTL were identified; two are associated with CBSD root necrosis only, and were detected on chromosomes V and XII, while seven were associated with CBSD foliar symptoms only and were detected on chromosomes IV, VI, XVII, and XVIII. QTL on chromosomes 11 and 15 were associated with both CBSD foliar and root necrosis symptoms. Two QTL were found to be associated with CMD and were detected on chromosomes XII and XIV, while two were associated with CGM and were identified on chromosomes V and X. There are large *Manihot glaziovii* introgression regions in Kiroba on chromosomes I, XVII, and XVIII. The introgression segments on chromosomes XVII and XVIII overlap with QTL associated with CBSD foliar symptoms. The introgression region on chromosome I is of a different haplotype to the characteristic “Amani haplotype” found in the landrace Namikonga and others, and unlike some other genotypes, Kiroba does not have a large introgression block on chromosome IV. Kiroba is closely related to a sampled Tanzanian “tree cassava.” This supports the observation that some of the QTL associated with CBSD resistance in Kiroba are different to those observed in another variety, Namikonga.

## Introduction

Cassava (*Manihot esculenta* Crantz.) is a staple food crop consumed daily by more than 800 million people, mainly in sub-Saharan Africa (Lebot, 2009). It is adapted to poor soils and harsh climatic conditions and is regarded as a food security crop, providing a yield when cereals and other food crops fail (Raphael, 2008). It is drought tolerant and offers a flexible harvesting regime since the roots can remain in the soil and be harvested when needed. Cassava is a commercial crop providing income particularly to women and the youth in the rural areas where it is sold raw or processed into various products. It is also used as an animal feed and is processed as starch for industrial purposes. Annually, approximately 229.5 million tons of cassava are produced worldwide with Africa contributing more than 118 million tons (51.4%) (FAOSTAT, 2015), which is greater than for any other crop in Africa. FAO statistics indicate the average yield of cassava in Africa to be 8 tons/ha, yet its potential yield is estimated to be 80 tons/ha (FAOSTAT, 2015).

Cassava yield in Africa is constrained by biotic and abiotic stresses. Cassava green mite (CGM), cassava mealybug, and the variegated grasshopper are the major cassava pests while cassava mosaic disease (CMD), cassava brown streak disease (CBSD), and cassava bacterial blight are some of the common diseases (Campo et al., 2011). Viral diseases are the most threatening and yield-limiting due to their current epidemiological and distribution trends (Legg et al., 2011). CBSD, originally considered to be localized mainly in the coastal regions of East Africa and limited by altitude below 1,000 m a.s.l. (Alicai et al., 2007), is now rapidly spreading to previously unaffected areas at alarming rates (Campo et al., 2011). It is thought that this is being fueled by changing climatic conditions, and the diversity and population dynamics of whitefly in the region (Legg et al., 2008). Conventional breeding is already underway in CBSD affected areas, but pre-emptive breeding is urgently needed in unaffected areas of Central and West Africa, the major cassava producing regions in Africa, to mitigate the threat posed by CBSD. The most feasible and sustainable control of cassava viral diseases is through the planting of resistant varieties that restrict virus multiplication and symptom development, thus limiting the spread and impact of the virus.

Cassava breeding for disease resistance, particularly CMD, began at the Amani breeding station in Tanzania in the 1930's (Hillocks and Jennings, 2003). After disappointing results from screening over 100 varieties sourced globally for CMD and CBSD resistance, the program focused on crossing the most resistant varieties and on inter specific hybridization (Nichols, 1947). Derivatives from *Manihot melanobasis* (now recognized as *Manihot* ssp. *flabellifolia*) were found to be very good candidates showing strong resistance with an ability to localize the virus at the base of the stem (Jennings, 1957). Crosses with *Manihot glaziovii* backcrossed three times and intercrossed with resistant hybrids produced interspecific hybrids that were rated over 80% resistant to CMD and moderately resistant to CBSD (Jennings, 1957). Some of the best known intercrosses at Amani included cultivars 5318/34, 46106/27, and 5543/156 (Jennings, 1994). The Amani breeding program was closed in 1958 and the materials that had been developed were distributed to many research centers in Africa. The Amani hybrids also became important for the International Institute of Tropical Agriculture's (IITA's) cassava breeding program in the 1970's at Moor Plantation in Nigeria (Beck, 1982). When the Amani program ceased, it is thought that some of the inter specific crosses found their way into farmers' fields in Tanzania and have been incorporated as farmer varieties (Kanju et al., 2003). The clones may have lost their identities and are being grown by farmers under different local names. Cassava breeders have identified some of these interspecific hybrids, and they show strong field resistance to CBSD. They are infected by the CBSD causative viruses; some show leaf symptoms, but the onset of root necrosis is delayed and limited allowing full yield potential (Hillocks and Jennings, 2003). These varieties are a rich genetic stock for cassava breeding, however, the genetic basis and mechanism of resistance are not clearly understood.

Kiroba is a farmer variety grown in coastal Tanzania (Muhanna and Mtunda, 2003) and is thought to be a former Amani hybrid that has lost its identity. Kiroba does not have any visible wild characteristics, shows mild CBSD leaf symptoms in Tanzania, but does not show any leaf symptoms in Uganda (Kaweesi et al., 2014), with almost no root necrosis even under very high disease pressure. A diversity assessment using SNP markers shows Kiroba to be quite closely related to a known Amani derivative, the landrace Namikonga (Ferguson et al., 2012). It is hypothesized that Kiroba may also be a derivative from a *M. glaziovii* × *M. esculenta* interspecific cross. These varieties have the potential to be utilized in cassava breeding programs to introgress CBSD resistance to farmer-preferred varieties or promising breeder's lines. The source and mechanism of their resistance and their pedigrees is not clearly understood. Genetic and genomic approaches are useful in identifying genomic regions that confer resistance in these varieties, information that is vital for the efficient movement of these traits in breeding, using marker-assisted selection (MAS) and genomic selection approaches.

Pre-emptive breeding measures for CBSD resistance are needed in West Africa, as current predictions indicate a high risk of disease spread to this region (Campo et al., 2011). Selection, in the absence of the disease, using molecular markers that tag resistance quantitative trait loci (QTL), has been used by International Center for Tropical Agriculture (CIAT) for CMD resistance in cassava (Okogbenin et al., 2012). Apart from facilitating selection in the absence of disease pressure, the application of molecular markers is expected to shorten the time for varietal development, a feature of conventional cassava breeding that is associated with a long breeding cycle and challenges of heterozygosity and outcrossing. Tagging QTL with molecular markers would also facilitate pyramiding of different resistance genes into a single variety, thereby promoting durability of resistance, and the spatial deployment of a diversity of resistance genes, if available.

Three genomic regions associated with CMD resistance have been recognized; CMD1 is a quantitative source of resistance identified in the Amani derived interspecific variety TMS 30572 (Fregene, 2000; Mohan et al., 2013), CMD2 is a region of large effect which has been widely used in IITAs breeding program and has been mapped in several studies (Akano et al., 2002; Lokko et al., 2005; Okogbenin et al., 2007; Rabbi et al., 2014), and CMD3 on the same chromosome as CMD2 (Okogbenin et al., 2012). Recently a genome-wide association study revealed the presence of an interacting locus, close to CMD2, which suggests either epistatic interactions or multi-allelic effects (Wolfe et al., 2016).

CGM resistance has been a target of conventional breeding in Zambia (Chalwe et al., 2015), however not much attention has been paid to this trait in East Africa, despite its importance, particularly in dry areas. Two SSR markers, NS1099 and NS346, have been linked to CGM resistance (Macea Choperena et al., 2012). SSR markers associated with early bulking, a physiological trait in cassava, have also been identified (Olasanmi et al., 2014).

The objectives of this study were to (1) identify QTL associated with resistance to CBSD induced root necrosis, CBSD foliar symptoms, CMD, and CGM, in a cross between Kiroba and AR37-80, (2) determine whether there are genomic regions in Kiroba introgressed from *M. glaziovii*, (3) determine if QTL lie within introgression segments, and (4) determine the relationship of Kiroba with other CBSD tolerant landraces based on whole-genome re-sequencing data. This would shed light on the origin of resistance observed in Kiroba and its relation with other CBSD resistant landraces in the region. The information is important for the deployment of a diversity of resistance genes for enhanced durability.

## Materials and methods

### Development of mapping population and phenotypic trials

The mapping population was formed by crossing two parents with contrasting responses to CBSD; Kiroba (female) and AR37-80 (male). Kiroba is a local landrace from Tanzania, which is largely male sterile, gets infected by cassava brown streak viruses (CBSVs), shows mild CBSD leaf symptoms with almost no root necrosis even under very high disease pressure and is regarded as CBSD tolerant by breeders (Kaweesi et al., 2014). In contrast, Kiroba is very susceptible to CMD. It is high yielding with a high dry matter content of over 30% (Muhana et al., 2003). AR37-80 is a CIAT improved variety being a cross between a CMD resistant line (C33) from IITA and CW259-42 which is a backcross of MTAI 8 (Rayong 60) and an interspecific cross between *M. flabellifollia* and CM 2766-5. It was developed through MAS, being positively selected for markers for the CMD2 resistance locus and markers for CGM resistance. It is resistant to CMD and CGM but susceptible to CBSD (Blair et al., 2007; Okogbenin et al., 2012).

Pairwise crossing blocks for Kiroba and AR37-80 parents were established at the Sugarcane Research Institute, Kibaha, Tanzania. At flowering [approximately 6 months after planting (MAP)] the female flowers from Kiroba were covered with pollination bags early in the morning (8–10 a.m.). The male flowers from AR37-80 with mature pollen were tagged at around 11 a.m., collected and hand pollination done at around midday. The pollinated flowers were tagged and after 4 weeks, the fertilized fruits were covered to avoid shattering. After 8 weeks, the seeds in the bags containing the tags were collected and stored for 1 month to break dormancy. The seeds were planted in trays and kept in a screen house. After 1 month the germinated seedlings were transplanted and maintained in a low CMD/CBSD pressure area of Makutopora (5°58′36.87″S, 35°46′00.00″E) in Tanzania in 2011. Stem cuttings were used to establish phenotyping trials in two coastal sites in Tanzania where CBSD disease pressure is high; Chambezi (6°33′19″S 38°44′51″E) and Naliendele (0°23′00.60″S, 40°09′50.58″E). Phenotypic trials and evaluations were conducted over two successive growing seasons, 2013–2014 and 2014–2015 using an alpha lattice design with incomplete blocks replicated twice. The F_1_ progeny were evaluated for CBSD leaf symptoms, CMD and CGM at 3, 6, and 9 MAP using a scale of 1–5 (Alicai et al., 2007). The roots were harvested 12 MAP and chopped into equal slices (5 cm) using a fabricated machine cutter. CBSD root necrosis was evaluated on a scale of 1–5 (Hillocks and Thresh, 2000). Seven roots per plant and a maximum of seven slices per root were chosen at random for disease scoring.

### DNA extraction and genotyping by sequencing

Genomic DNA was extracted from fresh young leaf tissues harvested from the F_1_ plants following a modified method of Dellaporta (Dellaporta et al., 1983). The F_1_ progeny were first screened to detect off types and selfs using 12 SSR markers (Kawuki et al., 2013) that were identified as being polymorphic between the two parents. Genotyping by sequencing (GBS) (Elshire et al., 2011) was performed on the confirmed progeny at the University of California, Berkeley (ICGMC, 2015). The SNPs were called using GATK (DePristo et al., 2011) against v5.1 of the cassava genome assembly and filtered using custom scripts. SNPs were named according to the chromosome number (Roman for v 5.1) and the base pair. Loci that deviated from the expected Mendelian segregation ratios based on goodness of fit (*P* < 0.05) were excluded from the analysis. Cluster analysis was performed with Mclust, grouping the samples based on identity by state (IBS) as a proxy for Identity by Descent (IBD) (ICGMC, 2015).

### Genetic linkage map construction

A genetic linkage map was calculated with SNP markers using the CP option of JoinMap v4.1 (Van Ooijen, 2006). A one-step map approach is the most common strategy for linkage mapping in cassava (Sraphet et al., 2011) and was used for this study. Goodness of fit between observed and expected segregation ratios was evaluated with the Chi squared test. Markers showing segregation distortion (χ^2^ > 6.0) were excluded together with identical (redundant) markers before calculating the linkage groups. Markers were grouped using the regression method at a minimum LOD threshold of 5. The recombination frequencies were converted into map distances (centiMorgans) using the Kosambi mapping function. The position of the markers in each linkage group were obtained by considering their contribution to the average goodness of fit (mean Chi square) and the nearest neighbor fit (N.N. Fit) value. Based on these criteria, markers were removed or added to each linkage group and calculations redone until the best fit and order was obtained.

### QTL analysis

Summary statistics for the phenotypic data were calculated using GenStat (Ripatti et al., 2009). The mean, skewness, kurtosis, and Shapiro-Wilk normality test were used to infer the distribution and normality of the data. Box plots and normal plots (Q–Q plots) were used to inspect the quality of the data and identify outliers. The mean of each genotype across the replicates in each year and site were calculated and used for QTL mapping. Inclusive composite interval mapping (ICIM) for QTL detection was done using the Genetic Analysis of Clonal F_1_ and Double cross populations (GACD) software (Zhang et al., 2015). A genome wide LOD threshold with P value of 0.05 was obtained by permutation test (1,000 replications) to identify significant QTL (Manichaikul et al., 2007). Additive (a) and dominance (d) effects were calculated based on the method of Muchero (Muchero et al., 2013);

$$\begin{array}{l} \text{a}=\left\{\text{mu}(\text{ac})-\text{mu}(\text{bd})\right\}/2;\\ \text{d}=\left\{\text{mu}(\text{ad})+\text{mu}(\text{bc})\right\}/2-\left\{\text{mu}(\text{ac})+\text{mu}(\text{bd})\right\}/2 \end{array}$$

where mu(ac) and mu(bd) are the phenotypic means for heterozygous loci carrying alleles derived from the same species and mu(ad) and mu(bc) are heterozygous loci carrying alleles derived from both species as computed by GACD software.

QTL mode of action was calculated as a ratio of dominance over additivity, d/a. According to Muchero (Muchero et al., 2013) d/a ratios of <1 are regarded as reflecting under-dominance, ratios between 0 and 1 reflect partial dominance while ratios >1 reflect over-dominance.

ICIM used in this study is an improved algorithm of composite interval mapping suitable for biparental crosses (Zhang et al., 2015). It has increased detection power, a reduced false detection rate, and less biased estimates of QTL effects. This approach minimizes the bias due to Beavis effect (Xu, 2003) associated with QTL analysis using a small population size and was suitable for our population that comprised of 106 individuals. QTL were named with q (for QTL), the name of the trait (e.g., CBSDRN for cassava brown streak disease root necrosis or CBSDRNF for root necrosis and foliar symptoms) followed by “c” for chromosome and the number of the chromosome on which the QTL lies, followed by “K” for Kiroba or “Ar” for AR37-80 e.g., qCBSDRNc11K. In cases where there was more than one QTL on a chromosome, an “L” or “R” was given as a suffix to indicate the left or right arm of the chromosome. When more than one QTL was present per chromosome arm, “a” and “b” were used to discriminate them.

### Re-sequencing

The main purpose of genome-wide re-sequencing was to investigate whether there are any genomic regions in Kiroba derived from *M. glaziovii*, investigate their genomic locations, and compare them with the identified QTL regions to infer the source of observed resistance. The varieties for this study were; *M. glaziovii*, a wild species of *Manihot*, also known as Ceará or India rubber, Albert, a Tanzanian variety said to be a pure *M. esculenta*, and Kiroba, one of the parents used in the mapping population reported in this study. DNA extractions, library preparations and sequencing was done as described in Bredeson et al. (2016). SRA BioSample accession numbers are as follows: Kiroba (SAMN02693378), Albert (SAMN04117017), *M. glaziovii* (SAMN02693380).

### Identification of genomic regions in kiroba derived from *M. glaziovii*

Sequence quality assessment was done using FastQC (Patel and Jain, 2012). The first 10 bases were trimmed using fastx trimmer and then *de novo* assembly performed using abyss-pe (Simpson et al., 2009). Default parameters were used with a k-mer of 64. The purpose of assembling the Kiroba genome was to obtain high quality scaffolds for alignment and SNP analysis. The quality of the Kiroba assembly was assessed by N50 length statistics derived from the abyss-pe output. Based on the results of the assembly, scaffolds and contigs smaller than 200 bp were discarded to avoid using low quality reads in subsequent analysis. Assemblies of *M. glaziovii* and Albert (*M. esculenta)*, were downloaded from Phytozome v.10 (Goodstein et al., 2012), and together with the Kiroba assembly, were aligned to v5.1 of the cassava genome assembly, using Bowtie2 (Langmead and Salzberg, 2012) followed by SNP calling using GATK.

### Calculating a score for possible introgression sites

The genotype information from the VCF file (the output of GATK pipeline discussed above) was coded as follows; 0/0 = 0, meaning homozygous to the reference, 0/1 = 1, heterozygous to the reference and 1/1 = 2, homozygous alternative. Any SNP with a missing value (−/−) for any genotype was removed. For each SNP, the absolute value for difference between the *M. glaziovii* and Kiroba (GK) and the difference between Albert and Kiroba (AK) scores were calculated. The final score for the SNP was determined using the equation: Diffscore = AK+ (2 − GK). Any SNP Diffscore value >2 was regarded as indicative of introgression. A loop was created to look at 1,000 bp at a time starting at position 1. The tail of the contig <1,000 bp was not analyzed. Poisson test was used to identify enriched regions of the genome, indicative of introgression (scores >2). The *p*-values were corrected for multiple hypothesis testing using a false discovery rate (FDR) with a 10% cut off to identify fragments that are significant and at least 1,000 bp.

### Comparative analysis of kiroba and namikonga haplotype derived from *M. glaziovii*

Chromosomal locations of introgression regions found in Kiroba were compared to those found in other genotypes by incorporating Kiroba into an earlier analysis (Bredeson et al., 2016). Comparative genome analysis was done to investigate whether Kiroba shared the same *M. glaziovii* haplotype as Namikonga and other genotypes including TME 117. Kiroba is thought to be a possible former Amani interspecific hybrid just like Namikonga and is postulated to have found its way into farmer fields and now being grown with unknown identity in Tanzania.

### Identification of genes present in the putative introgression regions and within the detected QTL regions

Putative introgression regions were aligned to the cassava reference genome v6 (Bredeson et al., 2016) using BLAST (Altschul et al., 1990). Gene lists containing functions and annotations were obtained from the top BLAST matches. Functional categories such as GO-Terms, PFAM domains, KOG, and PANTHER, were used for gene set enrichment analysis. Hypergeometric test with FDR correction cutoff of 10% (Benjamini and Hochberg, 1995) was performed to determine the significance of the functional term enrichment. The gene list for genes within the QTL regions and introgression segments were analyzed and tested for significance based on *P*-value and FDR.

### Genome-wide relatedness

Genetic relatedness analysis between Kiroba and 40 other accessions (Goodstein et al., 2012; Bredeson et al., 2016) including Namikonga and tree cassava was performed using the kinship coefficient $\hat{\pi }$, and identity by descent (IBD) probabilities computed with PLINK (Purcell et al., 2007). The SNP data for all the accessions was obtained from Phytozome v.10 (Goodstein et al., 2012) and filtered using Vcftools. A network plot showing the first degree relatedness was drawn using Cytoscape (Shannon et al., 2003).

## Results

### Development and validation of the mapping population

**Figure 1:**
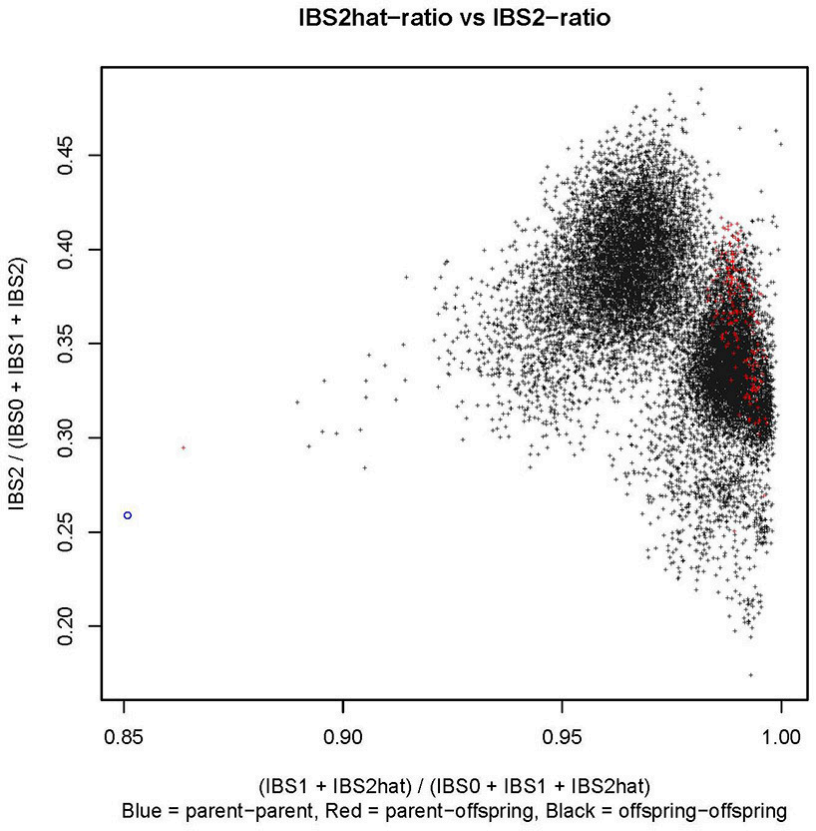
Identity by state (IBS) plot for the mapping population constructed using the GBS data. In the figure, parent–parent comparisons are represented by a blue circle, parent–offspring comparisons by red dots and offspring–offspring comparisons by black dots.

Controlled crossing through 1,116 hand pollinations between Kiroba and AR37-80 resulted in 2,676 seeds. Twelve hundred and sixty seeds were sown and 445 of them germinated in a screen house, a 35% germination rate. Three hundred F_1_ seedlings were transplanted in a CMD/CBSD free area in Makutupora, Tanzania, and only 280 of them survived in the field due to the hot, dry conditions. The mapping population was validated by SSR screening to identify off-types and true crosses. The 15 F_1_ progeny with unexpected alleles, presumably having received pollen from elsewhere were regarded as off-types and those with the expected allelic composition were classified as true F_1_. The integrity of the mapping population was further tested by cluster analysis of the GBS data based on identity by state (IBS) (Figure 1). The black dots on the figure represent offspring-offspring comparisons with full-sibs/F_1_ of the cross of interest clustering together toward the center of the plot (X, Y = 0.95, 0.35), selfs clustering near X, Y = 1.00, 0.25; and half-sibs producing clusters with X, Y = 0.90, 0.30. The parent–parent comparisons are denoted by blue circles and cluster around X, Y = 0.85, 0.25. The parent–offspring comparisons are denoted by red dots. Based on this analysis of GBS data, a further 75 off-types were identified as well as 205 true progeny. All 90 off-types were excluded from downstream analysis.

### Phenotypic trait evaluation and distribution

Summary statistics of phenotypic data obtained for two growing seasons 2013–2014 (year 1) and 2014–2015 (year 2) in two locations, Chambezi (C1 and C2) and Naliendele (N1 and N2) are presented in Table 1. The highest mean (3.073 of a maximum of 5) was obtained for root necrosis at C1 and the lowest was for CBSD foliar symptoms (1.032) in N1. The standard error of the mean (SE) ranged from 0.011 for CBSD foliar symptoms at N1 to 0.163 for root necrosis at C1. CBSD foliar symptoms were positively skewed toward class 1 in N1 and N2 (skewness 3.327 and 4.342). Root necrosis was also positively skewed in N1 and N2 (skewness 2.047 and 3.569). CGM was also positively skewed at C2. The highest variance was obtained for root necrosis in C1 (1.439) while the lowest was obtained for CBSD foliar symptoms in N1 (0.008). The SE followed a similar trend as the variance with the highest value reported for necrosis at CI and lowest 0.011 reported for CBSD foliar symptoms at N1. Kurtosis was highest in N2 for both CBSD foliar symptoms and root necrosis (20.891 and 20.619), respectively. The disease distribution across the sites for the same year and across years for the same site was significantly different for all traits measured *P* < 0.05 (Table 2).

**Table 1:** Descriptive statistics for phenotypic data for two years 2013–2014(1) and 2014–2015(2) for two sites, Chambezi (C) and Naliendele (N) in Tanzania.

Trait^*^	**Mean**	**Std. error**	**Std. deviation**	**Variance**	**Kurtosis**	**Skewness**	**Min**	**Max**	***P*-value**
*CGM_N1*	1.581	0.033	0.284	0.081	0.448	0.202	1.000	2.458	0.065
*CGM_N2*	1.978	0.038	0.315	0.099	1.536	0.597	1.200	3.000	0.075
*CGM_C1*	1.422	0.033	0.253	0.064	0.087	0.378	1.000	2.146	0.066
*CGM_C2*	1.103	0.025	0.206	0.043	6.560	2.543	1.000	2.000	0.051
*CMD_N1*	1.807	0.085	0.743	0.552	−0.193	0.811	1.000	3.778	0.170
*CMD_N2*	2.160	0.102	0.847	0.718	−1.139	0.146	1.000	3.900	0.204
*CMD_C1*	2.452	0.079	0.609	0.371	−0.837	−0.387	1.222	3.542	0.159
*CMD_C2*	2.413	0.087	0.709	0.503	0.130	0.184	1.000	4.300	0.174
*CBSD_Foliar_N1*	1.032	0.011	0.091	0.008	11.405	3.327	1.000	1.476	0.021
*CBSD_Foliar_N2*	1.151	0.050	0.416	0.173	20.891	4.342	1.000	3.600	0.099
*CBSD_Foliar_C1*	2.443	0.071	0.542	0.294	0.511	0.229	1.111	4.000	0.141
*CBSD_Foliar_C2*	1.510	0.044	0.358	0.128	−0.781	0.413	1.000	2.333	0.088
*CBSD_Necrosis_N1*	1.249	0.034	0.295	0.087	4.841	2.047	1.000	2.412	0.068
*CBSD_Necrosis_N2*	1.320	0.039	0.292	0.085	20.619	3.569	1.000	3.026	0.077
*CBSD_Necrosis_C1*	3.073	0.163	1.200	1.439	−1.068	0.066	1.042	5.000	0.327
*CBSD_Necrosis_C2*	1.916	0.100	0.788	0.620	−0.263	0.703	1.000	4.109	0.200

* *C1, Phenotyping in Chambezi year 1; C2, phenotyping in Chambezi year 2; N1, phenotyping in Naliendele year 1; and N2, phenotyping in Naliendele year 2. All traits are scored on a scale of 1–5 with 1 being no symptoms and 5 being maximum symptoms. P-value was calculated based on the 95% confidence limit*.

**Table 2:** Analysis of variance (ANOVA) across sites and years.

	Source of variation^*^	***SS***	***df***	***MS***	***F***	***P*-values**	***F*crit**
CBSD_Necrosis	N1_N2	1.3977286	1	1.3977286	3.194277255	0.0753386	3.886121
	C1_C2	10.487264	1	10.487264	4.800214211	0.0295564	3.886121
	C1_N1	25.4795	1	25.4795	14.55290527	0.0001792	3.886121
	C2_N2	8.9495113	1	8.9495113	10.26905208	0.0015636	3.886121
CBSD_Foliar	C1_C2	27.134009	1	27.134009	131.5793325	3.692*E*−21	3.918178
	N1_N2	0.5083999	1	0.5083999	5.792936116	0.0173668	3.907312
	C2_N2	4.3844347	1	4.3844347	29.02398243	3.118*E*−07	3.911795
	C1_N1	65.748541	1	65.748541	491.0939605	2.565*E*−46	3.912875
CMD	C1_C2	0.047749	1	0.047749	0.108293249	0.7426553	3.918178
	N1_N2	4.5239247	1	4.5239247	7.173528011	0.0082653	3.907312
	C2_N2	2.1493916	1	2.1493916	3.506911615	0.0633087	3.912331
	C1_N1	13.82904	1	13.82904	29.24131893	2.871*E*−07	3.912331
CGM	N1_N2	5.6987661	1	5.6987661	63.59393325	4.451*E*−13	3.907312
	C1_C2	3.1694071	1	3.1694071	60.03240176	2.985*E*−12	3.918178
	C1_N1	0.8384246	1	0.8384246	11.41468356	0.0009581	3.912875
	C2_N2	26.010994	1	26.010994	362.5913146	6.149*E*−40	3.911795

* *C, Chambezi; N, Naliendele phenotyping sites; SS, sum of squares; df, degrees of freedom; MS, mean square; F, F ratio; P-value, significance level; and F critical value set by the experiment. CMD denotes cassava mosaic disease and CGM cassava green mite*.

The Shapiro-Wilk normality test results are presented as Supplementary Table S1. CBSD foliar symptoms at C1 (P = 0.928 > 0.05), CGM in C1 and N1(*P* = 0.363 and 0.385), and CMD at C2 phenotyping year (*P* = 0.533) were normally distributed. The rest of the traits deviated from a normal distribution (*P* < 0.05).

### Genetic linkage mapping

**Figure 2:**
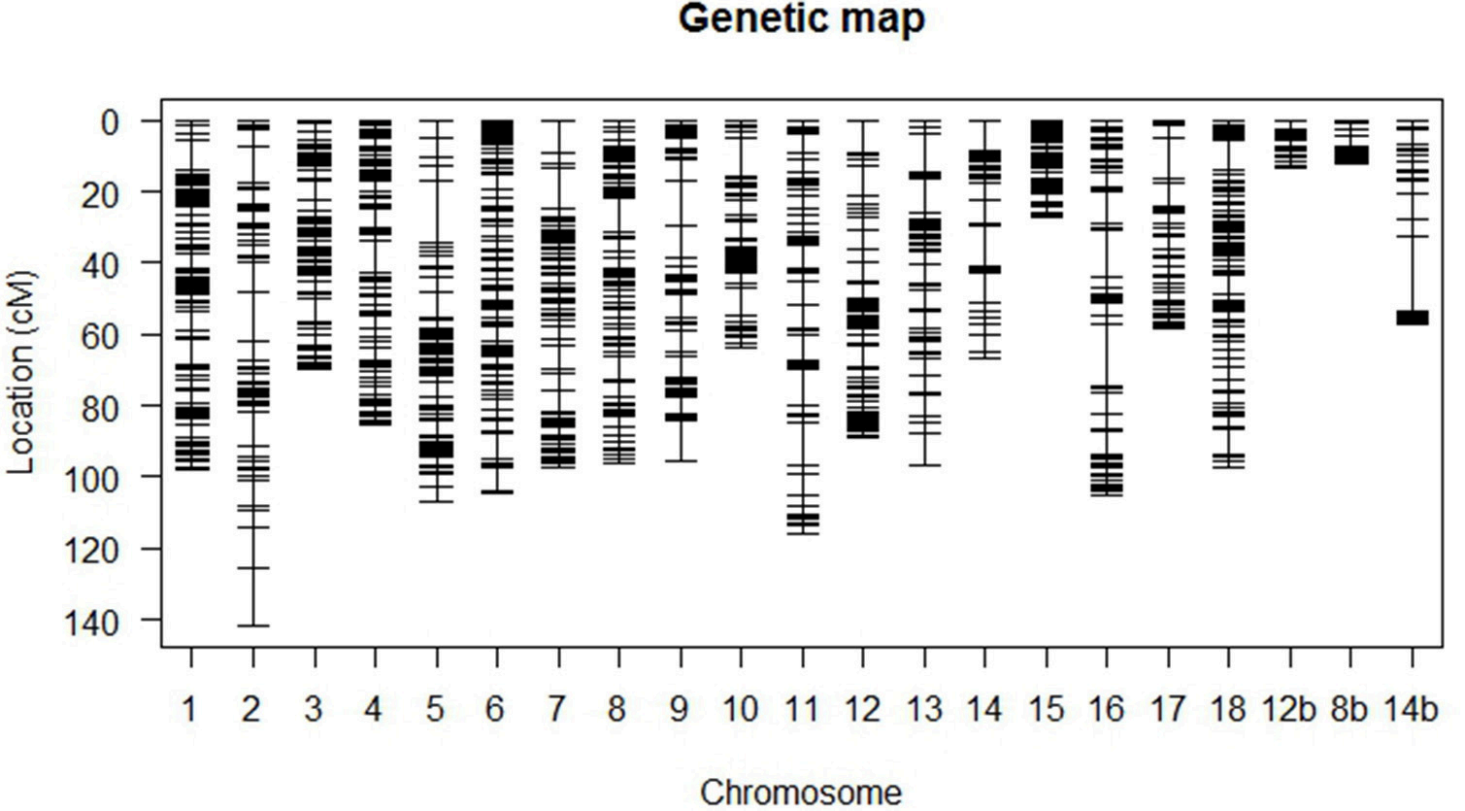
High-density genetic linkage map based on SNP markers. Linkage groups 1–18 represent chromosomes I to XVIII of cassava v5.1 assembly. Chromosomes VIII, XII, and XIV are represented by two linkage groups each and the second group is denoted by b.

A total of 4,422 SNP markers were found to be segregating in the mapping population with 3,873 markers conforming to the expected Mendelian segregation ratios and 549 markers (12%) showing moderate segregation distortion with 0.05 ≤ *P* ≤ 0.1. Some 1,548 (35%) markers were identical and excluded from the study leaving 2,874 unique markers (65%) for linkage analysis. The final linkage map (Figure 2) is composed of 106 F_1_ individuals with 1,974 SNP markers distributed across 21 linkage groups and spanning 1,698 cM. The 21 linkage groups represented the 18 chromosomes of cassava with chromosomes VIII, XII, and XIV each represented by two linkage groups.

### Significant QTL associated with CBSD, CMD, and CGM detected across environments

**Figure 3:**
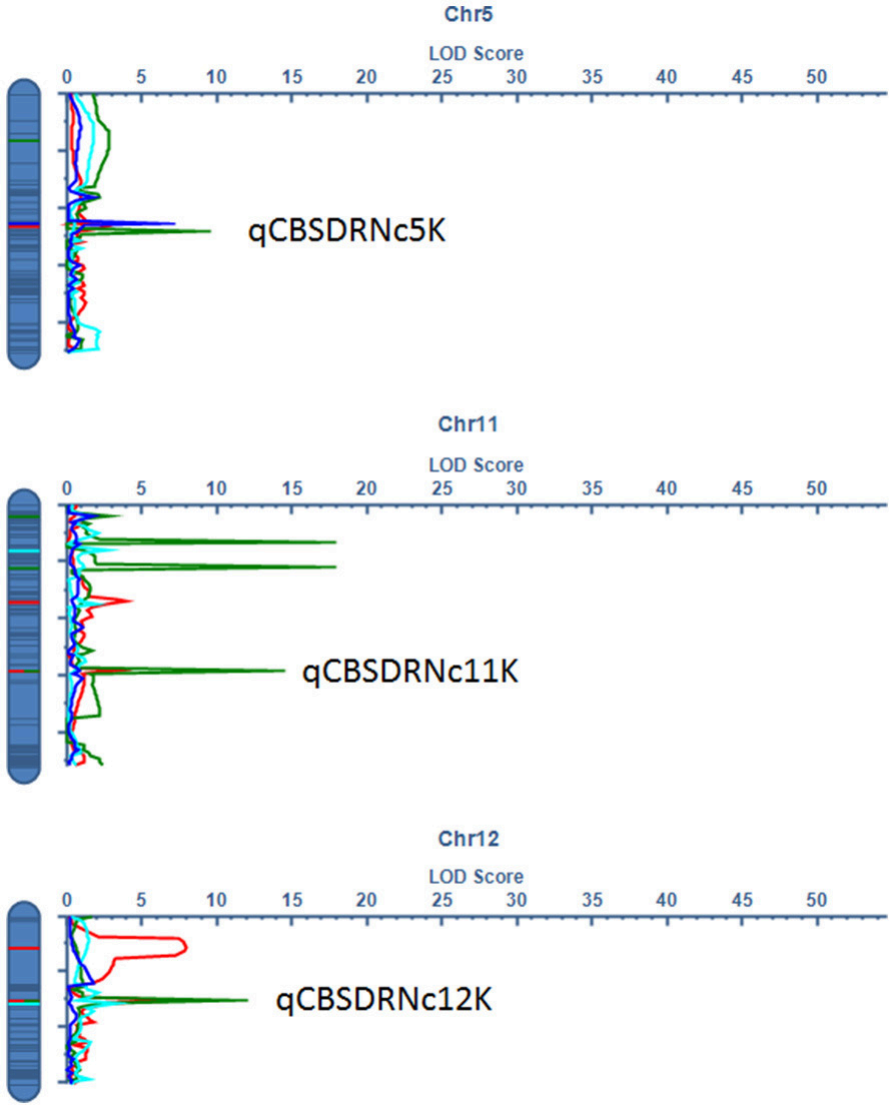
LOD profile for stable QTL associated with CBSD root necrosis at qCBSDRNc5K at C2 (green), N1 (red), and N2 (blue), root necrosis and foliar symptoms at qCBSDRNFc11K at N1 (red) and N2 (green), and root necrosis only at qCBSDRNc12K at C1 (sky blue), N1 (red), and N2 (green) detected on chromosomes V, XI, and XII, respectively.

Significant QTL were declared based on LOD threshold of above 2.5 and their stability across at least two environments (Table 3, Supplementary data 1). If the QTL was consistent across root necrosis and foliar symptoms in one or two environments only, it was declared a putative QTL, prefixed by “p.” Other putative QTL had closely located, but not identical, flanking markers. Based on these criteria, three QTL associated with CBSD root necrosis, namely qCBSDRNc5K, qCBSDRNFc11K, and qCBSDRNc12K, were detected on chromosomes V, XI, and XII, with maximum LOD-values of 6.20, 13.45, and 11.05, respectively, explaining up to 10.1% of the phenotypic variation (PVE%) (Figure 3). Seven QTL associated with CBSD foliar symptoms only, namely qCBSDFc4KL, qCBSDFc4R, qCBSDFc6KRa and b, qCBSDFc17K, qCBSDFc18Ka and b, were detected on chromosomes IV, VI, XVII and XVIII, respectively. They have maximum LODs of 2.78, 60.67, 54.75, 20.92, 27.01, 23.72 and 23.08, respectively, and explain up to 8.45% of the variation (Table 3). Two QTL were associated with both root necrosis and foliar symptoms, namely qCBSDRNFc11KR and qCBSDFc15K, although the latter was only associated with root necrosis in one environment (Naliendele 2014–2015). Two QTL, namely qCMDc12Ar and qCMDc14Ar, associated with CMD resistance were detected on chromosomes XII and XIV (Table 3), with maximum LODs of 13.20 and 4.41, and maximum PVE% of 13.01 and 13.36% of the variation. QTL associated with CGM, namely qCGMc5Ar and qCGMc10Ar, were detected on chromosomes V and X with maximum LODs of 20.19 and 24.03, and maximum PVE% of 10.56% and 10.08%. Seven putative QTL were also identified, five of these were associated with both CBSD root necrosis and foliar symptoms on chromosomes 4, 6 and 11 (Table 4). pqCBSDRNFc4KLa and b were closely located to one another on the genome and each occurred in one environment (N2 and C2 respectively) but for foliar symptoms and root necrosis. One QTL on the left arm of chromosome 6 (pqCBSDRNFc6KL) had inconsistent flanking markers for different traits, and on the right arm of the same chromosome, close to a consistent QTL, qCBSDFc6KRb, the putative QTL pqCBSDRNFc6KR, occurred in two environments (N1 and N2) but for one root necrosis and one foliar symptom trait only.

**Table 3:** Significant QTL detected in Kiroba × AR37-80 mapping population.

**QTL name**	**Trait**	**Location**	**Chr**	**Left Marker (v5.1)**	**Right Marker (v5.1)**	**LOD**	**PVE (%)**	**a**	**d**	**d/a**
qCBSDFc4KL	CBSD_3	C1	4	cIV:2397127	cIV:3389179	2.51	8.45	0.01	0.14	27
	CBSD_3	C2	4	cIV:2397127	cIV:3389179	2.78	4.19	−0.10	−0.22	2.15
qCBSDFc4KR	CBSD_3	N1	4	cIV:12722062	cIV:15281535	60.67	6.09	0.00	0.13	−
	CBSD_6	N1	4	cIV:12722062	cIV:15281535	20.77	7.38	0.01	0.42	72.95
	CBSD_6	N2	4	cIV:12722062	cIV:15281535	10.16	6.66	0.01	0.90	79.77
qCBSDRNc5K	Root necrosis	C2	5	cV:8584542	cV:9172040	6.20	10.18	−1.18	−1.14	0.96
	Root necrosis	N1	5	cV:8297662	cV:8525472	2.87	3.53	−0.34	−0.23	0.68
qCBSDFc6KRa	CBSD_3	N1	6	cVI:12787606	cVI:13554612	54.75	6.09	0.00	0.13	−
	CBSD_6	N1	6	cVI:12787606	cVI:13554612	24.12	6.49	−0.01	0.55	−60.99
	CBSD_6	N2	6	cVI:12787606	cVI:13554612	13.19	6.36	0.00	0.83	−
qCBSDFc6KRb	CBSD_6	N1	6	cVI:15579060	cVI:16110806	20.92	7.26	0.46	−0.49	−1.06
	CBSD_6	N2	6	cV1:15579060	cVI:16110806	3.04	2.64	0.32	−0.36	−1.13
qCBSDRNFc11KR	Root necrosis	N1	11	cXI:15686140	cXI:15799548	3.26	7.81	0.06	0.77	13.97
	Root necrosis	N2	11	cXI:15686140	cXI:15799548	13.45	3.12	−0.02	0.78	−34.69
	CBSD_6	C1	11	cXI:15686140	cXI:15799548	3.06	12.28	0.05	0.43	9.17
	CBSD_6	N2	11	cXI:15686140	cXI:15799548	13.54	6.40	−0.02	0.83	−36.39
qCBSDRNc12K	Root necrosis	C1	12	cXII:16811592	cXII:17259527	3.31	8.97	−0.15	0.44	−3.06
	Root necrosis	N1	12	cXII:16407934	cXII:16811592	7.42	8.02	0.03	0.78	27.57
	Root necrosis	N2	12	cXII:16407934	cXII:16811592	11.05	3.11	0.02	0.78	33.71
qCBSDRNFc15K	CBSD_3	N1	15	cXV:4187935	cXV:4668905	54.68	6.09	0.00	0.13	−
	CBSD_3	N2	15	cXV:4187935	cXV:4668905	32.68	4.49	−0.01	0.49	−55.14
	CBSD_6	N2	15	cXV:4187935	cXV:4668905	17.51	6.53	−0.05	0.85	−18.77
	Root necrosis	N2	15	cXV: 4187935	cXV:4668905	7.96	2.62	0.01	0.89	66.98
qCBSDFc17K	CBSD_6	C2	17	cXVII:18990126	cXVII:19117961	2.73	6.09	−0.68	−0.84	1.23
	CBSD_6	N1	17	cXVII:18990126	cXVII:19117961	27.01	7.39	−0.45	−0.51	1.14
qCBSDFc18Ka	CBSD_6	C2	18	cXVIII:5328493	cXVIII:5467691	2.96	7.17	0.00	0.75	−
	CBSD_6	N1	18	cXVIII:5328493	cXVIII:5467691	23.08	6.59	0.00	0.58	−
qCBSDFc18Kb	CBSD_6	C2	18	cXVIII:5764853	cXVIII:6089207	2.79	8.02	0.00	0.73	−
	CBSD_6	N1	18	cXVIII:5764853	cXVIII:6089207	23.72	6.59	0.00	0.58	−
qCGMc5Ar	CGM_3	C1	5	cV:8584542	cV:9172040	20.19	6.48	−0.40	−0.44	1.09
	CGM_3	N2	5	cV:8584542	cV:9172040	4.27	10.56	−0.17	−0.20	1.16
qCGMc10ArR	CGM_6	C2	10	cX:14135599	cX:15319838	24.03	4.11	−0.75	−0.79	1.05
	CGM_3	N1	10	cX:14135599	cX:15319838	3.83	10.08	−0.26	−0.33	1.27
qCMDc12Ar	CMD_3	C2	12	cXII:7399696	cXII:9335575	13.20	3.09	0.03	−0.72	−27.31
	CMD_6	C1	12	cXII:7399696	cXII:9335575	3.36	13.01	−0.15	−0.22	1.44
	CMD_3	N2	12	cXII:7399696	cXII:12974694	13.20	3.09	0.03	−0.72	−27.31
	CMD_6	N2	12	cXII:7399696	cXII:12974694	3.36	13.1	−0.15	−0.22	1.44
qCMDc14Ar	CMD_6	N1	14	cXIV:15032980	cXIV:16101978	4.41	13.36	0.82	−0.66	−0.80
	CMD_3	C2	14	cXIV:15032980	cXIV:16101978	3.54	1.22	−0.88	0.83	−0.94

*a, additive effects; d, dominance effects; d/a, QTL mode of action, all calculated according to Machero et al. (2013)*.

**Table 4:** Main Putative QTL in the biparental mapping population between Kiroba and AR37-80.

**Putative QTL name**	**Trait**	**Location**	**Chromosome**	**Left Marker (v5.1)**	**Right Marker (v5.1)**	**LOD**	**PVE (%)**
pqCBSDRNFc4KLa	Root necrosis	N2	4	cIV:4826000	cIV:8748959	17.57	3.12
	CBSD_6	N2	4	cIV:4826000	cIV:8748959	12.51	6.57
pqCBSDRNFc4KLb	Root necrosis	C2	4	cIV:8833268	cIV:8899624	2.64	8.46
	CBSD_3	C2	4	cIV:8833268	cIV:8899624	2.60	5.47
pqCBSDRNFc6KL	Root necrosis	N2	6	cVI:1126402	cVI:4229496	12.67	3.10
	Root necrosis	N1	6	cVI:7580997	cVI:8850841	6.22	6.83
	CBSD_3	N1	6	cVI:5913911	cVI:7196510	53.26	6.09
pqCBSDRNFc6KR	Root necrosis	N2	6	cVI:16482426	cVI:16676368	12.83	3.10
	CBSD_6	N1	6	cVI:16482426	cVI:16676368	12.15	7.38
pqCBSDRNc11KL	Root Necrosis	N1	11	cXI:7483911	cXI:7719727	3.94	2.81
	Root Necrosis	N2	11	cXI:4904507	cXI:5653088	16.90	3.15
	Root Necrosis	C1	11	cXI:2970283	cXI:4502176	2.69	17.75
	CBSD_6	N2	11	cXI:4156929	cXI:4502564	3.06	12.28
pqCBSDFc11KR	CBSD_3	C2	11	cXI:19060880	cXI:19576509	3.75	8.64
pqCGMc10ArL	CGM_3	N1	10	cX:5019671	cX:5019989	4.25	13.16
	CGM_3	C1	10	cX:5019989	cX:5188325	2.84	0.97

*C1, Chambezi field experiment 2013–2014; C2, Chambezi field experiment 2014–2015; N1, Naliendele field experiment 2013–2014; N2, Naliendele field experiment 2014–2015; PVE, phenotypic variation explained*.

A putative QTL region on the left arm of chromosome 11 (pqQTLRNFc11KL) had inconsistent flanking markers stretching from cXI:2970283 to cXI:7719727 but was present for root necrosis in three environments, and foliar symptoms in a single environment (Naliendele 2014–2015) (Table 4). The last putative CBSD foliar resistant QTL occurred on chromosome 11 at around 19Mb (Table 4). One putative QTL for CGM occurred on chromosome 10 but with inconsistent flanking markers.

### Additive and dominance effects

Five QTL, namely qCBSDFc17K, qCBCDFc4KL and -R, qCGMc10ArR and qCGMc5Ar, exhibited consistent evidence of over-dominance across sites whereas three QTLs, qCBSDFc18Ka and -b and, qCMDc14Ar under-dominance across sites (Table 3). qCBSDRNc5K showed evidence of partial-dominance. The rest of the QTLs were not consistent in their mode of action.

### Characterization of kiroba genome assembly

**Figure 4:**
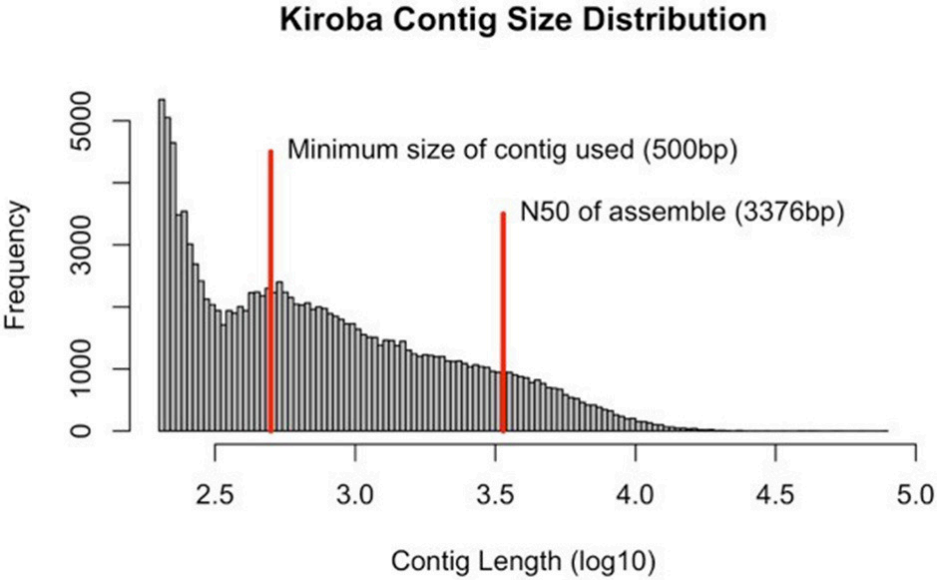
Kiroba genome assembly showing the contig size in log_10._ The minimum size used in the scaffold assembly was 500 bp (blue line) and the N50 (3,376 bp) of the contigs assembly (red).

Sequences were generated as paired-end reads from Illumina HiSeq 2500 and produced 310 million reads with 40X coverage (2 × 150 bp) of Kiroba, 257 million reads of 40X coverage of *M. glaziovii*, and 328 million reads with 42X coverage of Albert (*M. esculenta*) (Bredeson et al., 2016). The minimum contig size considered for *de novo* assembly into scaffolds was 500 bp. The N50 scaffold length for the Kiroba assembly was 3,376 bp (Figure 4). N50 length is the length of the smallest contig (scaffold) for which the collection of all contigs of that length or longer contains at least half of the sum of the lengths of all contigs. The final assembly had 13.83 million reads that were assembled into scaffolds whose sizes add up to 161.3 million bases. The percent coverage of the Kiroba assembly (using scaffolds >500 bp) used in this analysis was approximately 21.2% (161/760) of the *M. esculenta* genome (~760 million bases). The Kiroba genome assembly was of sufficient quality for comparative analysis.

### Genomic regions introgressed into kiroba and their co-location with detected QTL regions

**Figure 5:**
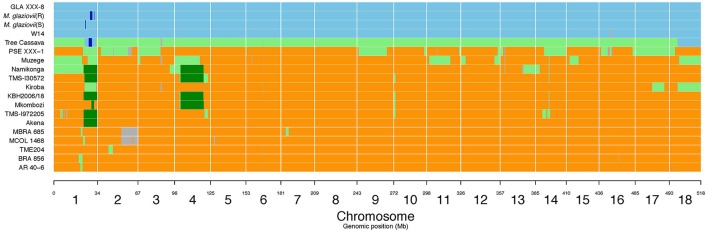
*M. glaziovii* introgression segments in selected cassava genotypes, including Kiroba and Namikonga, and based on whole genome sequence data. Orange indicates *M. esculenta* genotype; blue indicates *M. glaziovii*, light green represents hybrid *M. esculenta* and *M. glaziovii*, dark green indicates the presence of a shared haplotype proposed to be inherited from the Amani program (modified from Bredeson et al., 2016; Permission for the modification and publication of the adapted figure has been obtained from the copyright holder).

Large *M. glaziovii* introgression regions in Kiroba were found on chromosome 1 (24888098–33833841 bp), chromosome 17 (13700269–23436220 bp), and chromosome 18 (6805369–25209274 bp) (v6.1) of cassava genome (Figure 5). The QTL on chromosome 17, qCBSDFc17K (18990126–19117961 bp) and on chromosome 18, qCBSDFc18Kb (5764853–6089207 bp) associated with CBSD foliar symptoms lie perfectly within the large introgression regions, however qCBSDFc18Ka lies outside the introgression region. The QTL regions associated with CBSD root necrosis (qCBSDRNc5K, qCBSDRNFc11KR, and CBSDRNc12K), CMD (qCMDc12Ar and qCMDc14Ar), and CGM (qCGMc10Ar and qCGMc5Ar) are located on chromosomes 5, 10, 11, 12, and 14 that lack introgression regions derived from *M. glaziovii*. The introgression region in chromosome 18 encodes many protein domains that are associated with defense, including kinases, F-box family protein which contain leucine-rich repeats (LRR), tetratricopeptide repeat (TPR)-like superfamily protein, protein kinase superfamily protein, and pentatricopeptide repeat (PPR) superfamily protein.

### Identification of *M. glaziovii* haplotype present in kiroba and namikonga

Six genotypes, Namikonga, TMS130572, KBH2006/18, Mkombozi, TMS 1972205, and Akena, share a common *M. glaziovii* haplotype on chromosome 1 (Figure 5, dark green), designated as the “Amani haplotype” (Bredeson et al., 2016). Namikonga, TMS130572, KBH2006/18, and Mkombozi also share a common haplotype on chromosome 4 (Bredeson et al., 2016). The *M. glaziovii* haplotype in Kiroba is different from that of Namikonga and the other five genotypes that share the same “Amani haplotype” on chromosomes 1 and 5.

### Genome-wide relatedness

**Figure 6:**
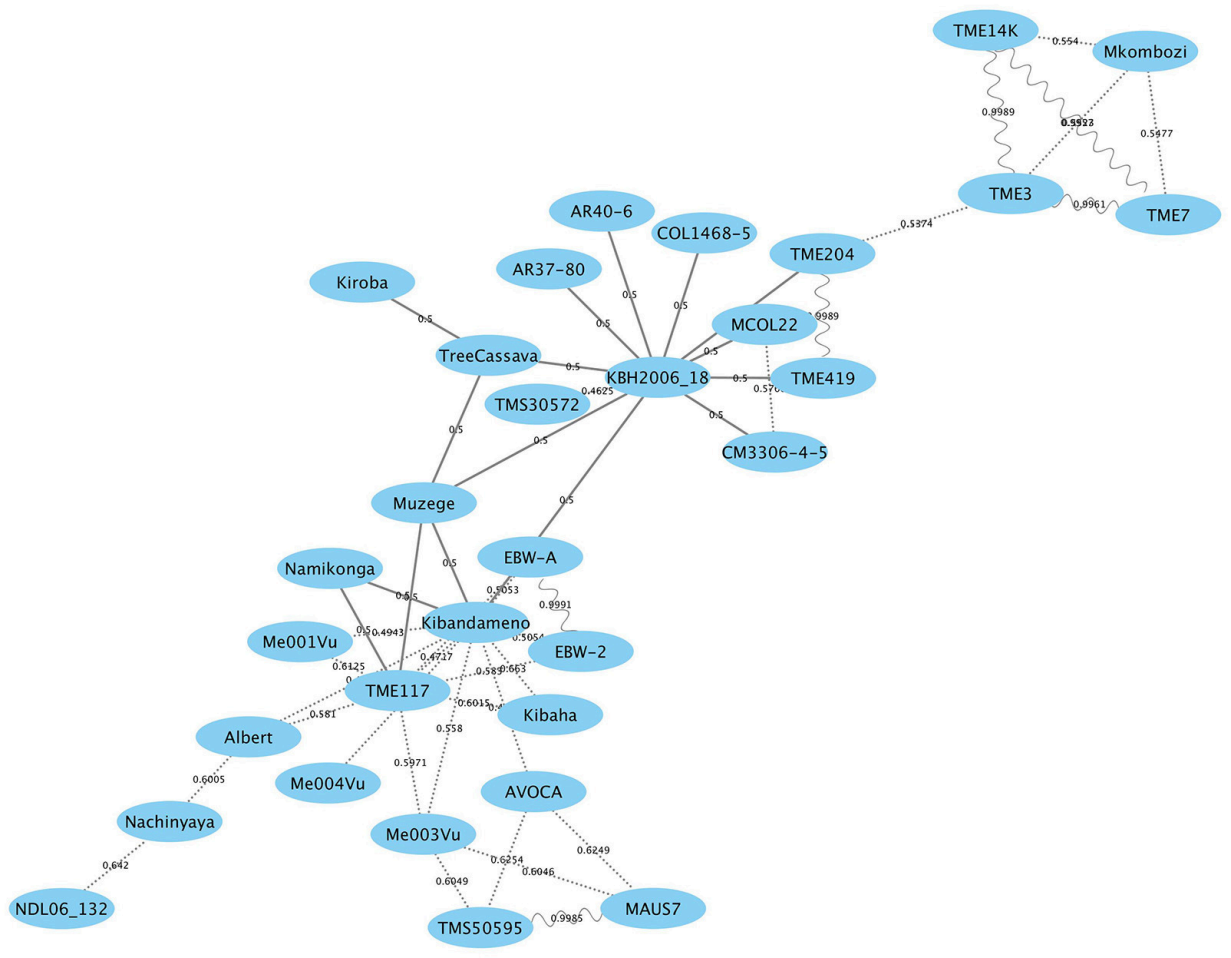
Genome-wide relationship between Kiroba and other genotypes with estimated kinship coefficients ($\hat{\pi }$) shown on the connecting lines. Parent–offspring relationship is represented by a solid line, full-siblings by dotted lines and identicals by sinewave (modified from Bredeson et al., 2016; Permission for the modification and publication of the adapted figure has been obtained from the copyright holder).

Individuals sharing putative first-degree relationships were identified as pairs sharing a $\hat{\pi }$ of 0.45 or greater (Supplementary Table S2). The results extend an earlier study (Bredeson et al., 2016) with the addition of Kiroba. A network of first degree relatedness (Figure 6) results shows two “hub” genotypes, namely TME117 and KBH 2006/18, each with many parent–offspring relationships. TME117 appears to be more related to East African germplasm such as Namikonga (with which it has a parent–offspring relationship), Albert, Ebwanatereka (EBW-A and EBW-2), and Kibaha, whereas KBH 2006/18 is more closely related to West African and South American germplasm such as TME204, TME419, AR40-6, and AR37-80. Kiroba, Muzege, and KBH 2006/18 have a parent–offspring relationship with tree cassava, with Muzege providing a link between the TME117 and KBH2006/18 hubs. Interestingly Kiroba appears to be more related to the KBH2006/18 hub than the TME117 hub that contains most of the East African germplasm.

### Genes with significant pest and disease resistance functional annotation terms within QTL regions

Significant terms and their associated annotations related to disease resistance within the QTL regions were obtained. They are significant by *P*-value and false discovery rate (FDR) correction. A total of 276 significant terms were detected within the QTL regions and 17 (6.2%) of them were related to pest or disease resistance in plants (Supplementary Table S3). 7.8% of the terms detected within the QTL regions for root necrosis are related to disease resistance. 3.3% of terms detected within QTL regions were related to CBSD foliar symptoms, 8% within QTL regions related to CMD resistance, and 3.3% detected within QTL regions related to CGM are associated with pest or disease resistance in plants. WRKY DNA-binding and leucine rich repeat (LRR) protein domains were found within QTL related to CBSD root necrosis on chromosome V and XII, while tetratricopeptide repeat (TPR) (chromosome VI), LRR (chromosome X), and F-box and pentatricopeptide repeat (PPR) (chromosome XVIII) are present within QTL regions associated with CBSD leaf symptoms. WRKY DNA-binding domains are present in the QTL region on chromosome V detected for CGM, while LRR and NB-ARC domains are present in the QTL regions detected for CMD resistance on chromosome XIV. The significant genes found on chromosome XI were not related to disease resistance in plants. There are only seven genes between the flanking markers of QTL on chromosome XVII, and none are particularly associated with disease resistance.

## Discussion

### Development of the mapping population

The mapping population used for QTL analysis was developed from a cross between Kiroba as the female parent and AR37-80 as the male parent. Kiroba is a local landrace found in coastal Tanzania and shows strong field resistance toward CBSD in that it gets infected by the viruses but shows no or minimal root necrosis and mild foliar symptoms even after two years under high disease pressure. Virus levels are kept low limiting the impact of the disease on yield (Kaweesi et al., 2014). Kiroba is susceptible to CGM and CMD. The male parent, AR37-80, is a CIAT bred line introduced to Tanzania to improve levels of dry matter, CMD, and CGM resistance in local germplasm (Blair et al., 2007; Okogbenin et al., 2012). Breeding in cassava is technically challenging due to its heterozygous nature, long growing cycle and low seed yield per pollination. It is highly outcrossing and difficult to develop an adequate sized F_2_ population usually limiting genetic studies to F_1_ progenies (Kunkeaw et al., 2010). An adequate number of seeds were obtained (2,676) however the germination rate was very low at 35%. This is likely to be due to the fact that seeds were germinated in pots on benches, exposing the soil to high diurnal temperature ranges. Placing the seed trays on the ground, thereby reducing diurnal temperature ranges that the soil and seed were exposed to, increased germination rates (results not shown).

SSR analysis and identity by state analysis of SNP data revealed patterns of relatedness, unrelatedness and some uncertainties of some of the F_1_ progeny. Some F_1_ progeny were assigned to different populations, as they were probably off-types, most likely having received pollen from a source other than from the donor parent during crossing. The number of progeny with unexpected alleles that were regarded as off-types was surprisingly high (90). This drastically reduced the number of genotypes available for linkage and QTL mapping in this study. Recent observations indicate female flowers may remain receptive for some time even after pollination and introduction of pollen from elsewhere may have been possible by other pollinating agents such as bees. The present assumption of non-receptivity of the flowers after pollination needs to change and the crossing technique modified to include bagging of the flowers even after pollination.

### Phenotyping analysis

CBSD disease distribution varied with site and season. CBSD foliar symptoms were higher in Chambezi for both phenotyping years; 2013–2014 and 2014–2015 (mean score 2.443 and 1.510) as compared to Naliendele (mean score 1.032 and 1.151). CBSD root necrosis was also higher in Chambezi for the two years (mean score 3.073 and 1.916) as compared to Naliendele (mean score 1.249 and 1.320). This shows that the CBSD disease pressure was higher in Chambezi as compared to Naliendele. All classes of scores (1–5) were represented in Chambezi for both leaf and root symptoms, but only classes 1–3 in Naliendele. The presence of different strains of virus in Chambezi or an increased population of whitefly may have contributed to this observation (Ndunguru et al., 2015).

The phenotypic data in this study generally do not follow a perfect normal distribution and this is not surprising as it is a common phenomenon observed in many mapping populations (Zou, 2009). CBSD foliar symptoms were positively skewed toward class1 in all the years in Naliendele (skewness 3.327, 4.342) unlike in Chambezi where the distribution tended to be normally distributed (skewness 0.0229, 0.413). This is supported by the kurtosis analysis showing Naliendele being positively skewed (kurtosis 11.405 and 20.891) and Chambezi minimally skewed indicating tendency for normality (kurtosis < 1 for all years). CBSD root necrosis follows a similar trend; Naliendele positively skewed (skewness, 2.047 and 3.569 kurtosis, 4.841 and 20.6190) and Chambezi minimally skewed with a tendency to be normally distributed (skewness and kurtosis < 1). This shows the disease pressure and distribution was high in Chambezi in all the years but much lower in Naliendele.

### Genetic linkage mapping

The genetic map was based on a one-step map approach (Rabbi et al., 2012). Bridge markers are needed in each linkage group for the maps to be integrated (Tang et al., 2015). The map is fairly saturated with 1,974 SNP markers (approximately one SNP every centiMorgan) and a maximum interval size of 21.4cM on chromosome XIV. The mapping population of this study contributed to the development of the consensus map (ICGMC, 2015). The consensus map is based on ten populations and contains 22,400 SNPs anchored in chromosomes and scaffolds of cassava version 4.1 assembly. The map obtained here is based on v5.1 genome assembly of cassava in which chromosome numbers are given in Roman numerals. In v6.1 of the genome assembly, Arabic numerals are used to designate chromosome numbers (Bredeson et al., 2016).

### QTL associated with CBSD resistance

Eleven QTL associated with CBSD resistance were identified using ICIM; two were associated with CBSD root necrosis and seven with CBSD foliar symptoms, and two with both foliar symptoms and root necrosis. These QTL were significant by LOD score (>2.5) and were detected in at least two environments. Given their relative stability, the QTL derived in this mapping population offer valuable targets for further characterization and utilization through genomic based approaches including marker-assisted breeding and genomic selection for improving cassava productivity. The QTL associated with root necrosis were found on chromosome V (qCBSDRNc5K located between 8.2–9.1 Mbp), XI (qCBSDRNFc11KR located between 15.6–15.7 Mbp), XII (qCBSDRNc12K located between 16.4–17.2 Mbp) and XIV (qCBSDRNFc15K located between 4.1 and 4.6 Mbp) (v5.1) with the highest LOD across years and environments in each case being 6.2, 13.45, 11.05 and 7.96, respectively. Interestingly the QTL associated with CBSD root necrosis, namely qCBSDRNFc11KR, at N1 and N2, mapped to the same interval and was flanked by the same markers as the one identified for CBSD foliar symptoms on chromosome XI detected at C1 and N2. This seems to suggest that CBSD root necrosis and CBSD foliar symptoms are influenced to some extent, but not exclusively, by the same gene(s), or by closely linked genes at this locus. This QTL region ranges from 21756682 to 21873545 bp on v6.1 of the cassava reference genome and lies within a CBSD resistance region (19872319–23751929 bp) (v6.1) on chromosome XI identified through genome wide association studies (GWAS) of populations in Uganda, partly derived from seed of Tanzanian origin (Kawuki et al., 2016). In those GWAS studies, four SNPs in this region were found to be associated with root severity and/or a disease index, and one SNP associated with maximum root severity. The fact that QTL and the QTL observed here in Kiroba are located in the same region indicates that Kiroba or close relative(s) sharing the same QTL may be the main source of resistance to CBSD root necrosis used in the Ugandan germplasm. qCBSDRNFc11KR is different from the QTL associated with CBSD root necrosis found in another Tanzanian farmer variety, Namikonga (Masumba et al., 2017). The QTL from Namikonga (qCBSDRNc11Nm) lies between cXI: 4502175 and cXI: 4760631, which is close to the putative QTL for root necrosis and foliar symptoms identified here in three environments (N1, N2 and C1) with flanking markers ranging from cXI:2970283 to cXI:7719727, but with two putative QTL (one for foliar symptoms) lying between 4.1 to 4.7Mbp (Table 4).

Five out of the 15 QTL identified were location specific, occurring in both years at one location (qCBSDFc4KL in Chambezi only; qCBSDFc4KR qCBSDFc6KRa and -b and qCBSDRNFc15K in Naliendele only). This could indicate genotype x environment interaction since our data show the disease distribution was not consistent across the sites. This may have also been influenced by the presence of different CBSVs strains in Chambezi and Naliendele experimental sites (Ndunguru et al., 2015). It is interesting to note that qCBSDRNFc11K was only found in Naliendele for root necrosis in both years, but was significant for foliar symptoms across locations.

### QTL associated with CMD and CGM resistance

QTL associated with CMD, namely qCMDc12Ar and qCMDc14Ar, were detected on chromosomes XII and XIV. qCMDc12Ar [7399696–9335575 bp (v5.1) equivalent to 7929400–8645361 bp (v6.1)] coincides with the major resistance locus (3.56–11.38 Mb) (v6.1) identified on chromosome XII (Wolfe et al., 2016) and confirmed as the QTL linked to the CMD2 locus (Akano et al., 2002). In that study a major peak was found at 7926132 bp. This is a major resistance locus that has been previously cited on scaffold 06906 and 05214 (Rabbi et al., 2014). The markers SSRY28 and NS158 that are linked to the CMD2 locus (Akano et al., 2002; Lokko et al., 2005; Fregene et al., 2006; Okogbenin et al., 2007) lie within qCMDc12Ar reported in this study. A CMD3 locus has also been reported on the same chromosome (Okogbenin et al., 2012) and is flanked by the marker NS198 which is approximately 36 cM away from the CMD2 locus. The presence of an additional interactive locus close to CMD2 has led to the postulation of multiple epistatic loci for CMD resistance or multiple alleles (Wolfe et al., 2016). The male parent of the mapping population studied here, AR37-80, was selected for CMD2 at CIAT using molecular markers (Blair et al., 2007; Okogbenin et al., 2007).

Most studies on CGM have focused on conventional breeding (Chipeta et al., 2013; Chalwe et al., 2015), with limited published work on molecular breeding (Macea Choperena et al., 2012). Two SSR markers, NS1099 (chromosome XIV) and NS346 (chromosome XVIII), showed high association with resistant families after screening five families each comprising 30 individual genotypes with 500 markers using bulk segregant analysis (BSA) (Ceballos et al., 2010; Macea Choperena et al., 2012). In this study we report two QTL linked to CGM resistance, namely qCGMc5Ar and qCGMc10Ar, on chromosomes V and X, with maximum LOD of 20.19 at C1 (with PVE 6.48) and 24.03 at C2 (with PVE of 4.11). These results, if validated and translated into marker-assisted breeding strategies, will complement conventional breeding approaches to improve cassava varieties for CGM resistance.

### Genomic introgression into kiroba and co-location with detected QTL

There are large *M. glazovii* like regions in chromosome 1 (24888098–33833841 bp), 17 (13700269–23436220 bp), and 18 (6805369–25209274 bp) (v6.1) of Kiroba (Figure 5) which is an indication of genomic introgression from this wild species. The co-location of qCBSDFc17K and qCBSDFc18Kb associated with CBSD foliar symptoms on chromosome 17 and 18 with the introgression segments suggest that resistance to CBSD foliar symptoms may be partly derived from *M. glaziovii*.

Approximately 7% of the Kiroba genome comprises an *M. glaziovii*/*M. esculenta* introgression segment, whereas in Namikonga approximately 14% of the genome has this form (Bredeson et al., 2016). Six genotypes (Akena, TMS-1972205, Mkombozi, KBH2006/18, TMS-I30572 and Namikonga) share a common *M. glaziovii* haplotype on chromosome 1 (Bredeson et al., 2016), and four genotypes (Mkombozi, KBH2006/18, TMS-130572, and Namikonga) also share a common haplotype on chromosome 4. These were designated as the “Amani haplotypes” (Bredeson et al., 2016). Kiroba does not share the so called “Amani” *M. glaziovii* haplotype on chromosome 1 or 4 that Namikonga shares with other genotypes. Unlike Namikonga, it does not have a direct relationship with TME117, but is more closely related to an interspecific hybrid “tree cassava” from Tanzania (*M. glaziovii* × *M. esculenta*), and the KBH 2006/18 hub (Bredeson et al., 2016). Tree cassava and Kiroba are related as parent–offspring according to the first degree relatedness analysis in this study. It is possible that tree cassava was used as a parent in the interspecific Amani breeding program, from where Kiroba is thought to have originated, as the rate of seed production in pure *M. glaziovii* × *M. esculenta* crosses can be very low (E. Kanju, pers. commun.)

This rather distinct relationship to the Namikonga-TME117-Nachinyaya-Albert-NDL06/132 cluster of germplasm (Figure 6) supports the finding of some different QTL associated with CBSD root necrosis and foliar symptoms, compared to those identified in Namikonga (Masumba et al., 2017). This would provide opportunities for pyramiding of QTL associated with CBSD resistance for more durable field resistance.

### Candidate genes in QTL regions

The QTL regions contain genes encoding several protein domains that have been reported to be involved in disease resistance in plants. The F-box protein domains found on chromosome XVIII are said to contain Leucine-Rich Repeat (LRR) domains associated with pathogen responses (Kuroda et al., 2002; Van den Burg et al., 2008). The pentatricopeptide repeat (PPR) superfamily protein (Barkan and Small, 2014) present in chromosome XVIII and tetratricopeptide repeat (TPR)-like superfamily protein (Prikryl et al., 2011) found on chromosome VI have been implicated in plant defense mechanisms. WRKY DNA binding domains found within QTL regions of chromosome V (associated with CGM and CMD) and XII (associated with CMD) are transcription factors involved in plant defense responses (Du and Chen, 2000). The F-box (Jakoby et al., 2002) and WRKY DNA binding domains (Eulgem et al., 2000) have orthologs in Arabidopsis with genes that function in plant defense. One percent of the total predicted genes in cassava contain these protein domains (Lozano et al., 2015) and have been shown to have high sequence similarity to proteins from other plant species, thus it is not surprising to find these genes in the QTL regions and within the putative introgression segments.

## Conclusions

This study has identified four QTL linked to resistance to CBSD root necrosis on chromosomes V, XI, XII, and XV, and these appear to be different from those found in the variety Namikonga (Masumba et al., 2017). Once validated, this would provide opportunities for pyramiding of QTL for root necrosis for enhanced resistance and greater durability. Nine QTL linked to CBSD foliar symptoms have also been identified on chromosomes IV, VI, XVII, and XVIII, with QTL on chromosomes XI and XV being consistent between root necrosis and foliar symptoms. This observation suggests that resistance to CBSD foliar symptoms may be controlled by some of the same loci to root necrosis, but additional loci are trait specific. A QTL associated with CMD resistance is consistent with the CMD2 locus on chromosome XII. Major *M. glaziovii* introgression regions are found on chromosomes I, XVII and XVIII, although the introgression region on chromosome I is not the characteristic “Amani haplotype”. The large introgression regions on chromosomes XVII and XVIII overlap QTL associated with CBSD foliar symptoms indicating that at least some of this resistance may be derived from wild species. All these QTL were detected using a small phenotyping population of 106 individuals and need to be validated on an expanded population size, to get greater accuracy. Once validated and applied through MAS or genomic selection, this study will aid in breeding cassava for multiple disease resistance, contributing to food security and enhanced economic growth in Africa.

## Ethics statement

All appropriate permissions have been obtained from the copyright holders of any work that has been reproduced in this manuscript.

## Author contributions

MF conceived, designed, and managed the research. IN, EM, FK, and KS collected phenotyping data. GM was project manager from Agriculture Research Institute, Tanzania, and facilitated the study through financial and logistical support. JB and MK provided training and technical support on introgression analysis. JL conducted GBS and JB conducted SNP filtering and calling under the guidance of DR. TS and IN conducted IBD analysis. IN constructed the genetic map and performed QTL analysis. SR provided scientific and bioinformatics support. IN and MF drafted the manuscript with scientific guidance from AM. All authors read and approved the article.

### Conflict of interest statement

The authors declare that the research was conducted in the absence of any commercial or financial relationships that could be construed as a potential conflict of interest.
