# Supplementary material for: QTL Mapping for Pest and Disease Resistance in Cassava and Coincidence of Some QTL with Introgression Regions Derived from Manihot glaziovii
Source: Front Plant Sci. 2017 Jul 21;8:1168. doi: 10.3389/fpls.2017.01168 (PMC5519584; doi:10.3389/fpls.2017.01168)
Supplement: Supplementary file 1 [file Table1.docx]

Supplementary Material

**QTL Mapping for Pest and Disease Resistance in Cassava and Coincidence of some QTL with Introgression Regions derived from *Manihot glaziovii***

Inosters Nzuki, Manpreet S Katari_,_ Jessen V. Bredeson, Esther Masumba, Fortunus Kapinga, Kasele Salum, Geoffrey Mkamilo, Trushar Shah, Jessica B. Lyons, Daniel S. Rokhsar_,_ Steve Rounsley, Alexander A. Myburg and Morag E. Ferguson^*^

*** Correspondence:** Corresponding Author: m.ferguson@cgiar.org

**Supplementary Table S1:** Shapiro-Wilk test for normality

| Trait | Test statistic W | Probability |
| --- | --- | --- |
| CBSD_Foliar_C1 | 0.9905 | 0.928 |
| CBSD_Foliar_N1 | 0.4117 | <0.001 |
| CBSD_Foliar_C2 | 0.9546 | 0.017 |
| CBSD_Foliar_N2 | 0.4078 | <0.001 |
| CGM_C1 | 0.9781 | 0.363 |
| CGM_N1 | 0.9824 | 0.385 |
| CGM_C2 | 0.5773 | <0.001 |
| CGM_N2 | 0.966 | 0.054 |
| CMD_C1 | 0.9548 | 0.028 |
| CMD_N2 | 0.9026 | <0.001 |
| CMD_C2 | 0.9836 | 0.533 |
| CMD_N2 | 0.9383 | 0.002 |
| Necrosis_C1 | 0.9554 | 0.043 |
| Necrosis_N1 | 0.771 | <0.001 |
| Necrosis_C2 | 0.9233 | <0.001 |
| Necrosis_N2 | 0.6512 | <0.001 |

**Supplementary Table S2:** Identical and first degree relationships

| **Identicals** |  |  |  |  |  |
| --- | --- | --- | --- | --- | --- |
| **Individual 1** | **Individual 2** | **IBD0 (Z0)** | **IBD1 (ZI)** | **IBD2 (Z2)** | ***π̂*** |
| EBW-2 | EBW-A | 0 | 0.0016 | 0.9983 | 0.999 |
| TME14K | TME3 | 0 | 0.0021 | 0.9979 | 0.999 |
| TME204 | TME419 | 0 | 0.002 | 0.9979 | 0.999 |
| MAUS7 | TMS50595 | 0 | 0.0029 | 0.9971 | 0.999 |
| TME3 | TME7 | 0 | 0.0077 | 0.9923 | 0.996 |
| TME14K | TME7 | 0 | 0.0085 | 0.9914 | 0.996 |
| **Likely parent -offspring** | |  |  |  |  |
| AR37-80 | KBH2006_18 | 0 | 1 | 0 | 0.5 |
| AR40-6 | KBH2006_18 | 0 | 1 | 0 | 0.5 |
| CM3306-4-5 | KBH2006_18 | 0 | 1 | 0 | 0.5 |
| COL1468-5 | KBH2006_18 | 0 | 1 | 0 | 0.5 |
| KBH2006_18 | Kibandameno | 0 | 1 | 0 | 0.5 |
| KBH2006_18 | MCOL22 | 0 | 1 | 0 | 0.5 |
| KBH2006_18 | Muzege | 0 | 1 | 0 | 0.5 |
| KBH2006_18 | TME204 | 0 | 1 | 0 | 0.5 |
| KBH2006_18 | TME419 | 0 | 1 | 0 | 0.5 |
| KBH2006_18 | TreeCassava | 0 | 1 | 0 | 0.5 |
| Kibandameno | Muzege | 0 | 1 | 0 | 0.5 |
| Kibandameno | Namikonga | 0 | 1 | 0 | 0.5 |
| Kiroba | TreeCassava | 0 | 1 | 0 | 0.5 |
| Muzege | TME117 | 0 | 1 | 0 | 0.5 |
| Muzege | TreeCassava | 0 | 1 | 0 | 0.5 |
| Namikonga | TME117 | 0 | 1 | 0 | 0.5 |
| **Likely full-sibling** | |  |  |  |  |
| Kibandameno | Me001Vu | 0.1745 | 0.6624 | 0.1631 | 0.494 |
| Albert | Kibandameno | 0.1731 | 0.6795 | 0.1474 | 0.487 |
| AVOCA | Kibandameno | 0.1508 | 0.7358 | 0.1133 | 0.481 |
| Kibandameno | TME117 | 0.1412 | 0.7742 | 0.0846 | 0.472 |
| Kibandameno | Me004Vu | 0.2212 | 0.6195 | 0.1593 | 0.469 |
| KBH2006_18 | TMS30572 | 0.1001 | 0.8749 | 0.025 | 0.463 |
| Albert | Nachinyaya | 0.0161 | 0.7668 | 0.2171 | 0.601 |
| Me003Vu | TME117 | 0.0027 | 0.8003 | 0.1969 | 0.597 |
| EBW-2 | TME117 | 0.0029 | 0.8283 | 0.1688 | 0.583 |
| EBW-A | TME117 | 0.0028 | 0.8284 | 0.1688 | 0.583 |
| Albert | TME117 | 0.0177 | 0.8027 | 0.1796 | 0.581 |
| CM3306-4-5 | MCOL22 | 0.0058 | 0.8347 | 0.1595 | 0.577 |
| Kibandameno | Me003Vu | 0.0836 | 0.7168 | 0.1996 | 0.558 |
| Mkombozi | TME14K | 0.0023 | 0.8874 | 0.1103 | 0.554 |
| Mkombozi | TME3 | 0.0014 | 0.8924 | 0.1061 | 0.552 |
| Mkombozi | TME7 | 0.0131 | 0.8784 | 0.1085 | 0.548 |
| TME204 | TME3 | 0.211 | 0.5031 | 0.2859 | 0.537 |
| EBW-2 | Kibandameno | 0.1308 | 0.7276 | 0.1416 | 0.505 |
| EBW-A | Kibandameno | 0.1309 | 0.7275 | 0.1415 | 0.505 |
| Kibaha | Kibandameno | 0.0194 | 0.6352 | 0.3455 | 0.663 |
| NDL06_132 | Nachinyaya | 0.0041 | 0.7078 | 0.2881 | 0.642 |
| AVOCA | TMS50595 | 0.004 | 0.741 | 0.2549 | 0.625 |
| AVOCA | MAUS7 | 0.0037 | 0.7428 | 0.2535 | 0.625 |
| Me001Vu | TME117 | 0.0026 | 0.7698 | 0.2276 | 0.613 |
| Me003Vu | TMS50595 | 0.0039 | 0.7824 | 0.2137 | 0.605 |
| MAUS7 | Me003Vu | 0.0035 | 0.7838 | 0.2127 | 0.605 |
| Kibaha | TME117 | 0.0028 | 0.7915 | 0.2057 | 0.602 |

**Supplementary Table S3:** Seventeen significant terms related to disease resistance, and candidate genes, found within the QTL regions

| **QTL** | **Term annotation** | **P-value** | **FDR adj P-value** | **Genes** |
| --- | --- | --- | --- | --- |
| qCBSDFc6K | K12200 programmed cell death 6-interacting protein | 3.77E-06 | 6.99E-06 | Manes.06G062600 |
| qCBSDFc6K | PTHR23083 Tetratricopeptide repeat protein, tpr | 0.001423 | 0.001748025 | Manes.06G062500 |
| qCBSDFc10K | KOG3017 Defense-related protein containing SCP domain | 7.80E-11 | 3.12E-10 | Manes.10G089800,  Manes.10G089900  Manes.10G090000 |
| qCBSDFc10K | K13449 pathogenesis-related protein 1 | 1.49E-06 | 1.99E-06 | Manes.10G089900 |
| qCBSDFc10K | PF08263 Leucine rich repeat N-terminal domain | 0.030015 | 0.034017167 | Manes.10G090500 |
| qCBSDFc10K | GO:0006952 defense response | 0.017246 | 0.032191706 | Manes.10G091500 |
| qCBSDFc18K | PF01566 Natural resistance-associated macrophage protein | 3.39E-07 | 4.41E-06 | Manes.18G080200,  Manes.18G080100 |
| qCBSDFc18K | PF00646 F-box domain | 4.10E-06 | 2.13E-05 | Manes.18G080900,  Manes.18G080800,  Manes.18G080700  Manes.18G080500 |
| qCBSDFc18K | PF01535 PPR repeat | 0.007722 | 0.01056644 | Manes.18G081800,  Manes.18G080300  Manes.18G081300 |
| qCBSDFc18K | PTHR22844 F-box and WD40 domain protein | 0.001121 | 0.001466151 | Manes.18G082000 |
| qCBSDRNc12K | PF03106 WRKY DNA -binding domain | 0.000512 | 0.000512491 | Manes.12G107400 |
| qCBSDRNc12K | PF08263 Leucine rich repeat N-terminal domain | 0.017957 | 0.020070087 | Manes.12G108300 |
| qCBSDRNc12K | PF01566 Natural resistance-associated macrophage protein | 1.55E-05 | 8.81E-05 | Manes.12G109500 |
| qCBSDRNc5K | PF03106 WRKY DNA -binding domain | 0.009542 | 0.011276814 | Manes.05G106900 |
| qCGMc5AR | PF03106 WRKY DNA -binding domain | 0.007003 | 0.008210781 | Manes.05G106900, |
| qCMDc14AR | PF00560 Leucine Rich Repeat | 0.065133 | 0.065132504 | Manes.14G161200  Manes.14G162700 |
| qCMDc14AR | GO:0006396 RNA processing | 0.005399 | 0.012675835 | Manes.14G164400 |
| qCMDc14AR | PTHR11017 Leucine-rich repeat-containing protein | 0.003087 | 0.003420248 | Manes.14G165100 |
| qCMDc14AR | PF00931 NB-ARC domain | 0.005761 | 0.006539726 | Manes.14G165100,  Manes.14G165300 |
| qCMDc14AR | GO:0006952 defense response | 0.013098 | 0.02720445 | Manes.14G165100,  Manes.14G165300 |
